# What makes a peer? Characteristics of certified peer recovery support specialists in an emergency department-based intervention

**DOI:** 10.1371/journal.pone.0289920

**Published:** 2023-12-07

**Authors:** Mia R. Kirk, Ashley D. Dawkins, Xing Wei, Olufemi Ajumobi, Lisa C. Lee, Roy Oman, Stephanie Woodard, Karla D. Wagner

**Affiliations:** 1 School of Public Health, University of Nevada, Reno, Reno, Nevada, United States of America; 2 Roots to Wings Consulting, LLC, Reno, Nevada, United States of America; 3 Division of Public and Behavioral Health, Bureau of Behavioral Health, Wellness and Prevention, State of Nevada, Carson City, Nevada, United States of America; University of Arkansas for Medical Sciences, UNITED STATES

## Abstract

Providing brief interventions by certified peer recovery support specialists (CPRSS) in the emergency department (ED) following a drug related visit is a promising method of service engagement and has garnered national attention. This study examines CPRSS’s perceptions of the qualities that enable them to deliver interventions in the ED. We conducted qualitative interviews with 14 CPRSSs working in EDs. Interview topics included how the participants became involved with CPRSS work, experiences working in the ED, how the ED differs from other settings, and what interactions with patients look like. Interviews were digitally recorded, transcribed, and analyzed for emerging categories. Three categories were identified relating to CPRSS work: (1) how they approach peer work, (2) inherent qualities required to do the work, regardless of the setting, and (3) context-specific skills required to do the work in the ED. When describing their approach to this work, participants talked about CPRSS work as their life calling and their passion. Participants also identified certain qualities that all CPRSS workers possess, regardless of the setting, including the ability to build rapport, strong listening skills, and a shared lived experience. Lastly, participants identified how specific hard and soft skills help them to navigate organizational and structural challenges in the ED. The unique conditions of the ED and the required qualities of a CPRSS should be considered when implementing an ED-based intervention.

## Introduction

Although there was a promising decline in overdose (OD) deaths in 2018 [1], the downward trend has reversed and the United States experienced an increase in OD deaths, from 65,000 in 2018 to over 100,000 deaths in 2021 [2]. Among people who inject drugs in North America, approximately 44.5% will experience a non-fatal OD in their lifetime [3] and many will be seen in the emergency department (ED). Surviving an OD increases the risk for experiencing a subsequent fatal or nonfatal OD [4]. Non-fatal drug-related visits to the ED have also increased dramatically since 2008 [5]. People who experience a non-fatal OD are less likely to engage with the healthcare system for routine medical care but more likely to visit the ED [6], and PWUD have higher rates of ED visits and hospitalization overall, compared to their non-drug-using peers. In a Canadian study, the odds of having two or more ED visits within a year were higher among PWUD who endorsed greater social and behavioral health needs, including receiving disability or income assistance and having mental health comorbidities [7]. Therefore, when someone presents to the ED for an OD or a drug-related concern, it is an opportunity to engage them in harm reduction services and link them to substance use disorder (SUD) treatment and other services, if appropriate. The ED has been identified as a crucial point of contact [8], described as both a “teachable” [9] and “reachable” moment [10] for PWUD.

Peer recovery-based support services are a strategy for boosting the recovery capital of people who use drugs (PWUD) while also addressing social needs such as housing, social connectivity, and employment [11, 12]. Peer support was first introduced in the mental health system as a method of person-centered, collaborative recovery [13]. Variations of the model have since expanded into substance use including smoking cessation [14], alcohol education [15], and most recently, post-opioid OD support in the ED [16]. Utilizing peer support in the ED following a drug related visit is a promising method of engaging PWUD and has garnered national attention [11].

A certified peer recovery support specialist (CPRSS) is someone with lived experience in successful recovery from substance use, mental health and/or other co-occurring challenges [17]. While certifications requirements vary by state, other qualifications include a high school diploma, GED, or higher; specialized training in delivering peer services (including multiple pathways to recovery, ethics, motivational interviewing, advocacy, crisis management, education, recovery capital, etc.); completing an internship, supervised experience hours, and examination; and agreement with a code of ethics [17, 18]. CPRSSs have been shown to empower clients, reduce hospitalizations, and make linkages to other resources [13]. CPRSSs may play a role in reducing the stigma associated with methadone treatment and improve retention for SUD treatment [19]. Moreover, interaction with a CPRSS in the outpatient setting has been associated with increased buprenorphine treatment engagement and opioid abstinence [20].

Some studies have examined the qualities that make CPRSSs effective in medical settings outside of the ED [19, 21–24]. Lennox et al., (2021) identified three characteristics of an effective peer worker in a hospital setting (but not specifically the ED): intrinsic qualities (such as being reliable, open-minded, respectful, adaptable, empathetic, sociable, and a good listener), contributions of shared experiences (acknowledging a past experience with substance use allowed the patient to feel more comfortable and understood), and personal stability (defined as an individual’s substance use pattern, mental health support, housing stability, and access to social supports). However, the ED is a unique clinical care environment in some ways that might require setting-specific adaptations. What is not known are the individual, organizational, and system level dynamics that allow for integration of CPRSS in the ED with the ultimate goal of improving patient outcomes.

In part because CPRSS models have been determined to be feasible and acceptable to health care providers working in settings such as the ED [8, 11, 25–28], CPRSS models have been rapidly implemented across the United States in hospital and community settings in response to the escalating opioid OD crisis [11, 19, 20, 27, 29]. It has also been shown that people who use opioids are open to and welcome engagement with a CPRSS in a setting such as the ED [16, 19]. Three perceived positive characteristics of a CPRSS identified by people who use opioids are the ability to offer empathy, encouragement, and hope during an ED intervention immediately following an OD [16]. Stewart, et al. (2021), identified five key features of ED-based interventions for OUD: patient identification; treatment approaches; program structure; relationship with community treatment programs; and program financing, sustainability, and maturity. Peer support services were described as “a key part” of these interventions, however a discussion on the characteristics of the peers themselves was missing [8]. Other studies point to what makes an effective workflow for CPRSS in the ED but not the unique characteristics of the people that make these interventions work [11]. Although these studies provide useful information on the need to and process for engaging CPRSSs in the ED, a formal investigation of the unique conditions of the ED and the qualities that enable CPRSSs to deliver an intervention in an ED is lacking.

The ED is a unique setting within the healthcare continuum. The ED is a fast-paced, unpredictable, and often overcrowded setting designed for acute clinical care [30, 31]. Staff are often under the pressure of budgetary constraints while also being asked to assume an increasing patient to provider ratio and greater responsibility [32]. ED staff are trained to attend to patients based on acuity, first caring for those with life threatening conditions. Although the immediate response to an OD or other substance use-related concern can be acute care, the underlying SUD is a chronic condition, requiring longer-term interventions including medications (e.g., to treat opioid use disorder), longer-term rehabilitation, social support, mental health referrals, and employment or housing assistance, all of which CPRSSs can facilitate [33].

Finally, all EDs have a unique culture [34] and generally operate under strict policies and protocols (e.g., restricted access areas, security, and parking) within a fairly hierarchical organizational structure [35]. This environment may demand unique characteristics and skills of the CPRSSs that are not necessary in other intervention settings such as in primary care offices, SUD treatment facilities, or in less-structured harm reduction-oriented settings.

Addressing the spectrum of SUDs and their health consequences requires a robust workforce of substance use and mental health professionals, of which CPRSS are one component. Although the evidence for peer-based interventions is accumulating [11, 12], less information exists about the unique characteristics and special skills that CPRSSs bring to their work in the ED. This study describes CPRSSs experiences in a ED-based intervention for people with SUD and characterizes the qualities of CPRSSs that they identified as important for their work.

## Material and methods

### Setting

In 2017, through funding from the SAMHSA State Targeted Response Grant, Nevada established post-OD outreach teams to provide interventions for patients presenting in EDs in the state. Although the intent of the program was to address non-fatal opioid ODs, soon after program implementation the CPRSSs responded to local need and expanded their scope to see patients who reported any substance use and behavioral health concerns. The teams are staffed with CPRSSs employed by community-based organizations. In Nevada, CPRSSs are trained by approved educational providers and certified through the Nevada Certification Board which adheres to the International Certification and Reciprocity Consortium (IC&RC) standards for CPRSS credentialing. The program is described in more detail elsewhere [16, 28]. At the time of data collection, CPRSSs were operating in two hospitals in the state. Initially the CPRSSs worked on-call and were dispatched when an eligible patient presented to the ED. Eventually, in both sites the CPRSSs were given space in the ED which they staffed 24/7. Based on the team’s monthly reports, the three most frequently reported substances were opioids, alcohol, and methamphetamine. The number of monthly referrals from the ED staff to the CPRSS teams ranged from about 10 in the first phase of program implementation (January 2019) to just under 100 in July 2022.

This study was part of a larger project designed to assess the feasibility, acceptability, and outcomes of an ED-based CPRSS intervention. The primary outcomes of the intervention are return visits to the ED, hospital admissions, and fatal OD. The larger mixed methods study included interviews with three constituent groups: (1) PWUD prior to intervention implementation to assess potential participants’ wishes for the program [16], (2) ED staff to assess feasibility of implementation [28], and (3) CPRSS to assess their experiences of the implementation (the current study).

### Participants

We interviewed 14 CPRSSs between January 2019 and June 2022 employed by three different community-based organizations. During that period there were approximately 23 CPRSSs employed across the agencies. However, the agencies experienced significant turnover during the study period which limited our ability to make connections with newly hired CPRSSs or contact CPRSSs who left the programs. We used convenience sampling and recruited participants by word of mouth and study flyers. Participants were contacted via telephone or email and informed of the study purpose and the role of the interviewer during recruitment and again during the informed consent process. Data collection ended when study staff agreed thematic saturation had been reached and all attempts to contact remaining potential participants were exhausted. All study procedures were approved by the University of Nevada, Reno, Institutional Review Board. We also obtained a Federal Certificate of Confidentiality from the National Institutes of Health.

### Data collection

One-time interviews were conducted one-on-one by full-time research study staff who have 5+years of qualitative research experience, lasted 30–90 minutes, and were conducted via Zoom or in person at a SUD treatment center or university affiliated office space. Only the participants and researchers were present during the interview. The study was granted a waiver of documentation of consent therefore no identifying information from participants was recorded. Verbal consent was obtained. The semi-structured qualitative interview guide was pilot tested and was not shared with the participants in advance of the interview. Participants received $50 cash for their expertise. Interviews were digitally recorded and transcribed verbatim by a professional transcription company and analyzed by three research staff members (First, second, and third authors). Interview notes were taken as needed after each interview. Transcripts were not returned to the participants for comment.

### Data analysis

Analysis was conducted from December 2021 to July 2022 using ATLAS.ti (Mac Version 8.4.5) [36]. Three analysts read interview transcripts in their entirety and derived codes from the data, based on the research question: “What qualities do CPRSSs believe are important for delivering a SUD intervention in the ED?”. Using Terry and Hayfield’s *Essentials of Thematic Analysis* [37] as a guiding framework, analysts read and coded each transcript individually then came together as a group after each transcript to discuss their findings. The initial coding structure consisted of index codes that closely aligned with the interview guide sections. This was a deductive process that allowed the analysts to begin their familiarization with the data. Subsequent analysis consisted of more “fine grain coding” where codes were assigned to smaller sections of any potentially relevant text based on patterns across transcripts, similarities or differences, metaphors, and indigenous typologies [38]. Memos documented the process of creating and defining codes, changes in analytic process, and how analysts arrived at an agreement on categories. Categories were derived by making meaning of the codes in relation to the entire dataset. Findings were also shared with coauthors and members of the research team to evaluate validity and disagreements were resolved through team-based decision making. Findings were not shared with study participants.

## Results

All participants (n = 14) were CPRSSs ranging in age from 26 to 62 years old. The majority of participants were white (64%), female (71%) and had worked in SUD treatment for an average of approximately 6 years (Table 1). Most participants had also worked as a peer in other settings including homeless encampments, areas where people use substances outdoors, SUD treatment facilities, and behavioral health agencies.

**Table 1:** Participant demographics (n = 14).

		n	%
Gender	Female	10	71%
	Male	4	29%
Hispanic or Latino ethnicity		4	29%
White race		9	64%
Mean age (SD; Range: 26–62)		43.6 (11.8)	
Mean years worked in substance use disorder treatment (SD; Range: 1–16)		5.9 (4.5)	

In the sections that follow, we discuss three primary aspects of doing CPRSS work in the ED (Table 2). First, we discuss the approach that CPRSSs take and the orientation they have to their work. Second, we describe the inherent qualities that the CPRSSs identified as being important for their work, in general. Finally, we will discuss context specific hard and soft skills that our participants believe are essential for doing CPRSS work in the ED, specifically.

**Table 2:** Categories related to certified peer recovery support specialist work in general, and in an emergency department setting, specifically.

Category title	Summary of category	Illustrative quotations
How CPRSSs approach their work.	A higher calling; their purpose, passion, life’s work; and more than just a job.	*“I don’t care if it’s just one person*. *It’s saving someone’s life*. *Letting them know that there is hope*.*”–CPRSS7* *“And so*, *when he asked me again why is it important that we do this*, *my answer was pretty simple*. *I do this for the same reason that most ER doctors and nurses got into this field*. *I’m doing this to save lives*.*”–CPRSS1*
CPRSSs possess inherent qualities, regardless of the setting.	Ability to build rapport, strong listening skills, a non-judgmental attitude, and a shared lived experience. Communication, compassion, and the ability to build trust.	*“I mean*, *just having a conversation*. *Um*, *not super formal*, *not stuffy*, *um*, *just having a conversation*. *And for the overdose thing*, *I mean*, *I’ve overdosed so many times*, *that I know how much it sucks*. *So*, *I think I’d probably launch into a conversation about that*. *And then be like*, *‘I’m so glad your friend found you” and then*, *‘man*, *you’re so lucky to be there right now*. *Glad we can have this conversation’*. *It would be along those lines*. *Rapport building*. *Not like*, *‘I am here to conduct an assessment’”*.*–CPRSS4*
CPRSSs in the ED employ context-specific skills.	Overcoming organizational and structural challenges and re-educating ED staff on the program. Hard skills: Technical expertise related to navigating the healthcare system including insurance, social services, and addiction medicine. Soft skills: Being assertive without overstepping boundaries with ED staff, the ability to adapt and be flexible to changing policies and the fast-paced environment, problem solving skills, being a team player, and identifying as a “people person.”	*Generally*, *when I can tell that somebody’s a little*, *like*, *tense or might not be telling me the full story or when I’ll be talking to the nursing department and hear that this patient told them one thing*, *but is telling me something different*, *I am trying to connect those dots*. *You know*, *a lot of people do have that shame and that fear of being judged*.*”–CPRSS12*

### How CPRSSs think about their work

When talking about their approach to ED-based work, broadly, most participants expressed the sentiment that this work is much more than a job, akin to their “life’s purpose”, a “higher calling”, and “life-saving” for themselves and others. This motivates them to continue the work, both formally in the ED and informally in other settings, despite personal challenges. They talked about an intrinsic motivation to do the work, which was often born from what they experienced before entering into their own recovery from SUDs. Participants believed that those experiences led to their calling of working as a CPRSS. For example, one participant relates her past life experiences as preparing her for her role as a CPRSS:

“*So*, *that’s basically how I got started [as a CPRSS]*, *I feel like it was my purpose and my reason for being here and going through all the things that I’ve been through in my life with fighting addiction for almost 3 decades*, *going in and out of jail*, *in and out of prison*. *For the last time*, *I knew that I needed to do something different and God placed in my heart that this was my purpose*. *This is the reason why he had been forming me all these years*, *for this purpose*, *peer support*. “–CPRSS9

Before becoming CPRSSs themselves, some participants had the experience of working with a peer and credit that experience with saving their life. “…*if it wouldn’t have been for peer support I probably wouldn’t be here today”* (CPRSS8). In turn, they see their role as a CPRSS as life-saving work. Other participants said if they had been given the chance to work with a peer when they were using substances, it may have positively altered their path and prevented them from traumatic events such as having their children taken away from them.

“… *when I was in active [drug use] stage*, *we didn’t have any programs like this or any services like this*. *And that’s something that would have helped me or I would have made an effort to try to utilize it*. *So*, *that’s why I love doing what I do now*.*”–*CPRSS11

In summary, participants see CPRSS work as their purpose in life and describe how the work transcends the limits of a job and has become their calling.

### CPRSSs possess inherent qualities, no matter the setting

Participants described important qualities inherent in all people who do peer work including rapport-building skills, strong listening skills, a non-judgmental attitude, and shared lived experiences. Participants described building rapport with the patients by “code switching” and not dressing too formally, so they would be distinct from the “white coats” and other hospital staff.

“*I’ve noticed peers*, *like*, *you code switch a lot in your language*, *right*, *like as the setting calls for it*. *So*, *somebody from the streets that’s cussing a lot like the peer might take that on and be like “Yeah*. *You know*, fu*k that.*” Like code switch*, *speak that lingo because it’s gonna build rapport……And then maybe you wouldn’t do that to like the senior citizen that’s like 78 years…You know*, *you’re probably gonna be like “Yes*, *yes*, *sir” or “Yes*, *ma’am” or whatever*. *And so*, *I think the interactions really depend on the person in front of you*.*”*- CPRSS3

While participants agreed unanimously that some form of lived experience is essential in allowing CPRSSs to relate to and build rapport with their clients, opinions differed on exactly how similar that lived experience needs to be. For one respondent, shared experiences of stigmatization, isolation, and discrimination were more important than experience using a particular drug:

“*I don’t really think the particular substance is so much of a difference as the fact that I was able to overcome the substance*, *which gives them hope that they too can overcome whatever substance whether it be opioids*, *whether it be methamphetamine or another stimulant*, *whether it be alcohol*.*”–*CPRSS10

Others believed that it was most useful if they had a similar history of substance use as the person they were supporting. However, some participants believed that while a shared history aided in the development of trust between the client and CPRSS, it takes more than a shared history of substance use to connect with someone. When asked what type of person would be successful in this position, they responded it’s necessary to be “good with people,’’ “an overall support system,” and “someone who really genuinely wants to help someone.”

Participants described incorporating some of the basic tenets of harm reduction into their work as a CPRSS, which helped with rapport building. By *“just meeting people where they are and walking with them to find a path that works for them”* (CPRSS3) and acknowledging with the patient that a “cookie cutter” or “one size fits all” approach doesn’t work for everyone. For example, one participant said, *“People cannot recover if they’re cornered into a method of treatment that does not resonate with them or if they’re dead or incarcerated”* (CPRSS12).

In summary, participants spoke of universal qualities such as communication skills, compassion, and the ability to build trust that CPRSSs possess for work in any setting, not just in the ED. By opening up to patients about their own lived experience, participants described the ability to quickly build rapport which allowed them to work alongside the patient on their unique journey, whether that was towards recovery at that time, or not.

### CPRSSs in the ED employ context-specific skills

Participants described the ED as a unique environment in two primary ways: (1) different levels of acceptance of the program and program literacy (e.g., a full understanding of what CPRSSs do and what benefit they can bring into the provision of care) [39] and (2) structure and organization of the ED (e.g., varying sizes, patient population, logistics, policies, and security protocols, time constraints, staff turnover and burnout, mistrust between provider and patient) that ultimately created a specific culture and impacted how they performed their work.

Participants described different levels of acceptance and literacy that the staff had about the program. For example, on one end of the spectrum, the CPRSS acknowledged the support from staff:

“*The thing that I really appreciated was that the hospitals—the nurses that did call*, *they empathized with the patient*. *They felt for ‘em*. *They wanted to get them the help*. *They wanted to go the extra mile*. *So*, *that made it all worthwhile*. *I think that with them being in that position especially at the ER*, *it’s very busy*. *And for them to take out the time to call and say ‘hey*, *this person needs help*, *they’re going through different changes and they said they wanted to get some help so now we’re calling you’—So*, *it takes a lot for them to have to stop whatever they were doing to make this call to keep calling over and over until they finally get me ‘cause sometimes they can’t reach me right away because I might be in the office with a client and then for them to turn around and call back*.*”–CPRSS9*

On the other end of the spectrum, some participants experienced pushback from ED staff who either felt they just did not have the time to engage with the CPRSS or expressed stigmatizing attitudes that created barriers to providing holistic patient care.

“*They [ED staff] were more bothered than anything that they would have to call peer support or the mobile outreach unit*. *They would rather just discharge the person and put them right back on to the streets than have to take the time to treat them*.*”–*CPRSS8

“*They [ED staff] definitely have some stigma*. *They feel burdened by these patients*. *It’s almost like they feel that they shouldn’t be at the hospital*. *Um*, *because as soon as they identify substance use*, *it doesn’t matter what else they might have come in for*, *they’re only associating them with their substance use”*.–CPRSS6

Another aspect of the context specific nature of the ED that affects how CPRSSs do their work is structural and organization issues such as staffing. For example, some EDs have high staff turnover and others rely on travel nurses who do short-term assignments, which required ongoing work from the CPRSSs to ensure that new ED staff are aware of the program.

In addition, the workflow and culture of EDs varies significantly by location, patient load, and patient population. CPRSSs who worked in multiple EDs noted that each one has its own culture, including the degree of program literacy (i.e., a full understanding of what CPRSS do and what benefit they can bring into the provision of care) [40].

“*Um*, *but they said that they try to get people in and out as quickly as possible because they only have [a limited number of] beds*, *so they really don’t want them sitting around waiting for somebody*. *They just want to treat them and get them out*. *But*, *they have a lot of high utilizers and it’s become a problem*. *And so*, *most of the clinicians were really curious about adding a process as a way to prevent some of the high utilizers presenting with these symptoms*, *related to substance use*.*”* -CPRSS4

In response to these challenges, participants identified hard and soft skills required to work in these settings. Hard skills included technical expertise related to navigating the healthcare system including insurance, social services, and addiction medicine. Soft skills included being assertive without overstepping boundaries with ED staff, the ability to adapt and be flexible to changing policies and the fast-paced environment, problem solving skills, being a team player, and identifying as a “people person”.

Participants navigated the structural challenges such as frequent turnover by providing periodic training for new ED staff, showing up to “team huddles”, and in general “making their presence known”.

“*I used to go to = Hospital name = as well*. *I used to go to their huddles [i*.*e*., *ED staff meetings] just making my presence known to remind them I’m here*. *If somebody needs help*, *call us*, *you know*. *So*, *that’s basically what my thing was as far as staying in touch with them and having a rapport with them is just meeting them every week for their meetings*.*”-CPRSS9*

Eventually, participants were able to work with the ED staff to develop best practices and once the program was established at the ED, they were often seen as experts and became a routine part of the patients’ care.

“*the first words out of [ED physician] mouth when he walks in the room and has one of these situations*. *He’s like “Call her*. *We don’t have to deal with this*. *We have an expert who’s an expert in this field*.*” Not an expert*, *but knowledgeable in this field and can help*. *Why do we want to stand here and try to figure out what this guy needs when somebody else can do it and we can go take care of other patients*?*”*-CPRSS1

In addition to being a “people person”, CPRSSs also perceived knowledge of the larger system and how to navigate it as an essential trait for their work in the ED. The system of care for people beginning their recovery journey is complex and often involves a series of referrals between treatment organizations, healthcare providers, insurance companies, government agencies, social services, and more. The CPRSS may have progressed through this same system of care and is able to problem-solve with the client and facilitate an entrance into the most appropriate care, if the patient desires. For example, one participant described the multiple steps it takes to find an appropriate treatment option for a patient:

“…*And from there*, *I make some calls*. *And based on availability and insurance*, *I do try and find placement that is like all-encompassing*. *Most people that I see*, *there is like a behavioral health component*, *so somewhere that is able to provide that clinical assessment for them and is able to give them that like one-on-one counseling*, *and identify maybe those underlying conditions that are making it so hard for them to establish sobriety*, *and making sure that that is facilitated for them as well*. *And once I find that best fit that lines up with all those components*, *I’ll have them sign release of information to send the hospital’s paperwork over to that facility for screening purposes and one for the facility that they’re going out to so that I can call and follow-up on their treatment once they’re there*.*”–*CPRSS12

Participants were also able to identify when patients may be slipping through the cracks and work to prevent that with future patients. Further, the CPRSSs spoke of maintaining professional boundaries, being mindful of the jurisdiction of care and also fostering a learning opportunity for ED staff to understand the CPRSS-patient relationship. In general, the complexity of the ED requires tact and mindfulness to navigate the environment.

“*I’ll come back [to the ED] and I’ll follow up ‘Oh*, *whatever happened with so and so*? *I heard this case worker was gonna be determining placement*.*’ And then I find out that the patient is discharged*, *you know*. *And they’re just ‘Oh*, *well*, *they were sent to a homeless shelter because the social worker is per diem and they tried to call this place*.*’ The ball kind of does get dropped from time to time*. *We try and smooth that over as much as we can*, *and we’ll get whatever contact information we can*. *But you know*, *if we’re not right on top of it*, *if we follow-up too frequently*, *they’ll get annoyed with us*. *But if you don’t follow-up like within just the right amount of time*, *you find out that*, *oh*, *this person was just sent out to a homeless shelter because they called one facility*, *and they were full*.*”* -CPRSS12

## Discussion

ED-based CPRSS-delivered interventions for PWUD are being rapidly scaled up across the US, and evidence regarding their effectiveness is beginning to accumulate [29]. Our findings contribute to this growing body of literature by highlighting the nuances that shape how CPRSSs do their work in an ED setting compared to other settings such as general medical, inpatient hospitals, or community-based services. We found that CPRSSs consider this work a higher calling that they are driven to do. There are inherent qualities that they possess no matter what setting they are working in, and there are context-specific hard and soft skills that CPRSSs employ when working in an ED setting.

Participants in our study described that their approach to their work is shaped by an inherent sense of purpose and passion, often derived from their own experiences as people in recovery from SUDs. A “sense of purpose” and other reinforcers (such as employment, financial rewards, social support, and enjoyable experiences) have been proposed as critical components of sustained recovery from SUD and other challenges [41, 42]. In fact, the act of helping others or giving back as an act of service is foundational to some recovery pathways, including those that adhere to 12-step principles. Our findings suggest that working as a CPRSS can provide such reinforcement to some people (through providing a sense of purpose and meaningful employment), which could both improve patient outcomes and help sustain long-term recovery for the CPRSSs themselves. However, it is also important to recognize that CPRSSs may experience negative individual level dynamics such as occupational stress, burnout, compassion fatigue, and vicarious trauma through their work. Ensuring that organizations employing and supporting CPRSSs have high-quality, recovery-centered supervision is critical to improve training, retention, and quality of services delivered.

The ED is a unique context for providing interventions, and our participants suggested that both global and specific skills are required of CPRSSs in this setting. Soft skills, sometimes referred to as personality traits, are more challenging to assess than hard skills (like knowledge of the healthcare system), however are often equally as valued in the workplace [43]. The degree to which they can be learned versus are innate lies on a spectrum [43]. Globally, CPRSSs must have good rapport building skills, strong listening skills, and a non-judgmental attitude. These findings map well onto the IC&RC CPRSS certification Domains: (1) Advocacy, (2) Mentoring and Education, (3) Recovery and Wellness Support, and (4) Ethical Responsibility [44]. The value of a shared lived experience, of having “walked in their shoes”, was also identified as a critical component that distinguished CPRSSs from other providers of behavioral or social support, similar to findings from other research [41]. One unique finding from our study is that participants were not unanimous in their assessment of *which* experiences must be shared. While some people thought that a history of having a shared drug of choice was essential, others disagreed. The degree to which, and on what attributes, peers and patients should be similar in their lived experiences is an area for future research.

Additionally, working with both patients and professionals from a variety of disciplinary backgrounds and specialized training such as nurses, social workers, physician assistants, and medical doctors placed CPRSSs in the position of having to “code switch” as a way of engaging in ingroup identification, identity management, and distancing behaviors. Turner et al. (1987) [45] describes ingroup identification as an important component of social cohesion. Ingroup solidarity is associated with a high degree of commitment to their group and a strong sense of identity [46]. Scholars have noted that individuals in recovery often draw on organizational narratives that place an emphasis on treatment services for PWUDs as a technique to manage one’s own stigmatized identity [47, 48]. Based on the narratives that discussed the precarity of managing one’s identity within this setting by switching communication styles as well as the heavy emphasis on connecting individuals to detox, treatment, and formalized services rather than offering harm reduction strategies, further research could focus on the relationship between self-identity, cohesion in the recovery community, and whether the CPRSS’s recovery journey influences the services to which ED patients are connected.

Experiences of the CPRSS in our study were both similar to and different from the perspectives of ED staff. In line with other research among ED clinicians examining feasibility and acceptability of ED-based intervention programs [28], participants spoke of the potential benefits to the patients (by adding additional resources) and ED staff (by freeing up clinical resources and reducing their workload) conferred by adding a CPRSS program to the ED.

Other research has suggested that medical professionals on the front lines of the opioid OD epidemic are experiencing challenges including inadequate knowledge and training about SUDs, limited resources, and negative emotions directed at patients with SUDs [49]. As identified by our participants, these conditions can be exacerbated by high turnover of clinical staff, especially when understaffed hospitals rely on temporary travel nurses or other health care professionals [50]. According to our findings, integrating CPRSSs into the ED may help to develop best practices and provide expertise about SUDs that was otherwise lacking in the ED, thereby supplementing, and providing education for the clinical staff.

In research with patients, CPRSSs have been described as potential patient advocates who can help buffer the more negative experiences with some ED clinicians [16]. Furthermore, the unique attributes identified by our participants may help with the specific challenges of working with a patient who has just overdosed. For example, based on their shared lived experience, CPRSSs may be able to empathize with that patient better than a clinician who does not have the experience of overdosing. Additionally, the CPRSS can exercise their best judgement on what type of support to provide and to what depth. Knowing that the time immediately following an OD can be emotionally and physically challenging and that all patients are at different levels of willingness to change their behavior, the CPRSS may act as a confidant by primarily listening and sharing their story. Or they may provide referrals, complete paperwork, and drive the patient to their next stage of care. Although integrating a CPRSS program into an ED could help mitigate some challenges associated with caring for patients with SUD, care must be taken to ensure that the emotional burden is not shifted entirely to CPRSSs, which could jeopardize their own wellbeing. This reinforces our previous recommendation to ensure that CPRSSs have access to quality peer supervision that is recovery-focused and adequately addresses individual needs.

## Future research

As the field moves forward with research on the effectiveness of ED-based CPRSS programs, there is a need to more precisely understand the essential elements of an intervention in the ED including dose, provider, and desired outcomes [11]. Related to dose, questions remain related to how long the CPRSS needs to meet with the patient while in the ED and how frequently post-discharge to be most effective. In terms of the intervention provider, a recent study found high rates of linkage to SUD treatment within 30 days from an ED-intervention, and found no difference based on whether the intervention was delivered by a CPRSS or a social worker [29]. However, more qualitative research is needed to understand the experiences of providing and receiving ED-based interventions from CPRSSs compared to other providers, which can inform more robust outcomes analysis. Future research can focus on the patient perspective after meeting with a CPRSS including how that experience impacted their time in the ED, subsequent substance use, and other outcomes. Further, given the well-documented increase in burnout among emergency medical providers associated with the opioid epidemic, research should examine the impact of providing these services on CPRSSs’ wellbeing. Lastly, by definition, CPRSSs are in recovery from substance use, mental health, or co-occurring disorders. Although this quality is considered essential, it may also create barriers to engagement in other harm reduction-oriented services that do not prioritize abstinence. There is a risk that peers could perpetuate SUD stigmatization of clients whose behaviors and priorities around substance use do not match their own [19].

## Limitations

This study is not without limitations, largely related to generalizability and transferability. First, we used convenience sampling to recruit participants and were unable to contact all the eligible CPRSSs, which could introduce selection bias. Due to turnover within the organizations we were unable to reach some participants and cannot assess differences between our sample and non-respondents. Second, this study was conducted within two hospital systems in Nevada, a large, mostly rural Western state, and findings may differ from research in more urban areas with a more robust health care infrastructure. Third, the intervention and data collection began during the COVID-19 pandemic, which resulted in significant challenges to implementing the program in the EDs and may have resulted in fewer peers participating in this study. Fourth, the CPRSSs in Nevada are employed by a community-based organization (not the hospitals) with a harm reduction orientation. While there are benefits to the CPRSSs being employed by a harm reduction, community-based organization, our findings may differ from the experiences of peers employed by the hospitals or health care system.

## Conclusion

ED-based interventions for people with SUD are rapidly scaling up across the US in response to an unprecedented number of opioid OD deaths. Although evidence for the effectiveness of peer-based programs exists in other settings, the ED is unique and may require some special adaptations and qualities to ensure program success. We found that CPRSSs working in the ED setting identified some global qualities, such as relationship building, communication skills, and shared lived experiences, but also some context-specific attributes that CPRSSs perceive as important for their work with PWUD. CPRSSs represent promising agents for the delivery of effective SUD interventions. Understanding what barriers exist at recruiting, training, and retaining this workforce is an important next step in delivering ED-based interventions with the ultimate goal of improving outcomes for PWUD.
